# Effects of Short‐Term Breathwork on Respiration and Cognitive Performance in a Discrimination Task

**DOI:** 10.1111/ejn.70737

**Published:** 2026-11-04

**Authors:** Lena Hehemann, Lisa Stetza, Christoph Kayser

**Affiliations:** ^1^ Department for Cognitive Neuroscience, Faculty of Biology Bielefeld University Bielefeld Germany

**Keywords:** behavior, breathwork, cognitive performance, respiration, respiratory dynamics

## Abstract

Respiration is a unique physiological process that can operate automatically but can also be deliberately used to modulate the bodily state. Importantly, respiration is deeply intertwined with neural processes, influencing functions from arousal and attention to executive control. Although structured respiration (breathing practice) has been exploited for centuries, the immediate consequences of short periods of structured respiration for sensation and cognition remain poorly understood. The present explorative study examined how brief, 1‐min episodes of structured respiration affect respiration and performance in a subsequent sensory‐cognitive task. Across two experiments, participants engaged in structured breathing practices that manipulated either respiratory frequency (slow breathing vs. fast breathing) or the inhalation–exhalation ratio (short inspiration–long expiration and long inspiration–short expiration) prior to performing a visual emotion discrimination task. Immediately after breathing practice, participants' respiration deviated from their baseline: Each technique resulted in specific deviations of respiratory frequency, inhalation–exhalation ratio, or the occurrence of atypical respiratory cycles, suggesting technique‐specific returns to regular respiration. During the subsequent discrimination task response accuracy and reaction times did not differ between breathing practices. However, we observed transient changes in reaction times immediately following all practices, suggesting a brief facilitation of attentional or sensorimotor responsiveness following conscious breathing. Our findings indicate that even brief, consciously controlled respiration can transiently influence cognitive performance, highlighting the role of voluntary respiratory modulation in shaping brain function and behavior.

## Graphical abstract

Different respiration practices transiently influence reaction times in an emotion discrimination task, demonstrating that active modulation of respiratory dynamics can shape cognitive performance.

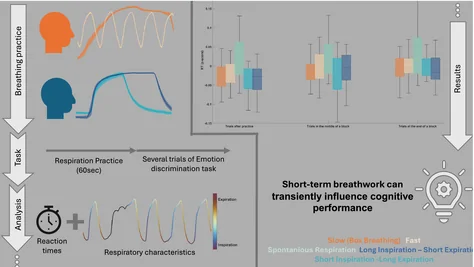

**Abbreviations**
BF_10_
: Bayes factor (evidence for the alternative hypothesis relative to the null)
CO_2_
: carbon dioxide
EEG: electroencephalography
FCRs: fraction of correct responses
FDR: false discovery rate
HPA axis: hypothalamic–pituitary–adrenal axis
HRV: heart rate variability
Hz: Hertz
I/E ratio: inhalation‐to‐exhalation ratio
LISE: long inspiration–short expiration
ms: milliseconds
NOI: no intervention
O_2_
: oxygen
RT: reaction time
SD: standard deviation
SILE: short inspiration–long expiration

## 1 Introduction

Respiration is more than a metabolic necessity; its rhythm is deeply embedded in the brain's activity, influencing processes from arousal and attention to executive functions. While the effects of breathing practices (i.e., active breathwork) on well‐being, stress and emotional regulation are well documented (Chin et al. 2024; Fincham, Kartar, et al. 2023; Fincham, Strauss, et al. 2023; Kral et al. 2023; Röttger et al. 2021; Russo et al. 2017; Vanutelli et al. 2024; Zaccaro et al. 2018), their specific impact on cognitive performance has only recently received systematic attention. A growing body of research has demonstrated that during spontaneous, unstructured respiration performance in sensory‐cognitive tasks systematically covaries with the respiratory cycle. Participants tend to actively adapt their respiratory pattern to the temporal structure of laboratory tasks, with direct consequences for immediate task performance and subsequent memory performance (Brændholt et al. 2025; Della Penna et al. 2026; Flexman et al. 1974; Grund et al. 2022; Huijbers et al. 2014; Perl et al. 2019; Saltafossi et al. 2025). For example, across a large sample of laboratory tasks spanning discrimination and detection paradigms and a memory task, we have shown that both reaction times (RTs) and the accuracy of perceptual reports covary systematically along the respiratory cycle, in a manner that renders the respiratory phase shortly prior to a response informative about the response speed and response accuracy (Harting et al. 2025; Johannknecht and Kayser 2022). Similar respiration phase–dependent effects have been observed in emotional—but not visual (Gabor patch)—discrimination tasks (Mizuhara and Nittono 2023) and in memory retrieval, where performance varies depending on whether respiratory transitions occur from inhalation‐to‐exhalation (I/E) or vice versa (Nakamura et al. 2018). These behavioral effects are paralleled by changes in large‐scale rhythmic brain activity, functional connectivity, and local markers of task‐related attention in sensory and association cortices (Brændholt et al. 2025; Folschweiller and Sauer 2021; Kluger et al. 2021; Liu et al. 2017; Maric et al. 2020; Perl et al. 2019; Stetza et al. 2025; Tort et al. 2018, 2020; Zelano et al. 2016) possibly driven by the extensive projections of brainstem respiratory centers to higher order brain structures (Ito et al. 2014; Yackle et al. 2017) and the strong connectivity of the olfactory system with the limbic cortex (Nguyen Chi et al. 2016; Yanovsky et al. 2014; Zelano et al. 2016).

This intricate connection between respiration, brain activity, and cognitive performance provides a strong theoretical basis for how active breathwork could enhance perceptual and general cognitive performance. Indeed, one finds recommendations for structured or mindful respiration across social media, healthcare literature and much more. Controlled respiration encompasses various techniques that supposedly induce distinct physiological and psychological effects, both following short‐term application and long‐term practice. For example, practicing slow deep breathing has been shown to enhance parasympathetic tone and vagal activity (Pal and Velkumary 2004), to foster sleep quality (Andas et al. 2023), and to improve general health markers and subjective ratings of well‐being (Balban et al. 2023; Rahmathulla et al. 2024). In combination with simple exercise, a 10‐week deep breathing program was shown to reduce stress, mood disturbances, heart rate, and cortisol levels (Perciavalle et al. 2017). But also, short‐term breathing practice can enhance autonomic function, mood, and cognition. Studies show that slow breathing increases heart rate variability (HRV), a physiological marker of reduced stress (De Couck et al. 2019), and promotes faster RTs to auditory stimuli (Gallego and Perruchet 1993). Hyperventilation or high‐frequency yoga breathing, in contrast, were associated with immediate analgesic effects (Chalaye et al. 2009), enhanced emotional well‐being (Van Diest et al. 2014), increased cortical activity, attention‐related processes (Telles et al. 2024; Budhi et al. 2024), and improved RTs to visual and auditory stimuli (Neginhal et al. 2017).

The possible pathways through which breathing practice could shape cognitive functions are multifold. Many practices induce changes in heart rate and its variability (De Couck et al. 2019; Van Diest et al. 2014; Neginhal et al. 2017; Sroufe 1971). They can thereby alter vagal tone, which can enhance cognitive control, attentional regulation, and emotional stability via activation of the sympathetic nervous system (Smith et al. 2017; Thayer and Lane 2000, 2009). Studies also link breathing practices, such as slow breathing, to systematic changes in brain rhythms such as alpha band activity (Budhi et al. 2024; Telles et al. 2024). This is associated with attentional processes and arousal (Hanslmayr et al. 2011), suggesting that respiration‐induced rhythmic neural activity may reflect one pathway through which voluntarily modulated respiration could directly influence sensory‐cognitive processing. Moreover, diaphragmatic and deep breathing have been shown to reduce cortisol secretion (Ma et al. 2017; Martarelli et al. 2011; Perciavalle et al. 2017), underscoring their modulatory effect on the hypothalamic–pituitary–adrenal (HPA) axis, a key neuroendocrine system implicated in the regulation of cognitive functions (Erickson et al. 2003; Wolf 2003).

Despite the general consensus that structured respiration exercises can have a positive influence on subjective well‐being and physiological and cognitive functions, it remains unclear which specific respiratory patterns are most beneficial and whether (or which) benefits can be obtained even after short periods of structured respiration. This lack of understanding comprises two questions: First, it remains unresolved whether potential positive effects of breathing practice on sensory‐cognitive performance are driven by changes in respiratory frequency, the temporal structure of inhalation and exhalation, or unspecific effects related to intentionally controlling respiration. Second, whether conscious breathing over a time scale as short as 1 min, which could be easily integrated into many everyday situations, would provide benefits for sensory‐cognitive performance. Here, we address both of these. Benefits of long‐term breathwork are well documented. Understanding the potential effects of short‐term breathwork, however, is important both to pave the way to everyday‐applicable strategies and to establish laboratory paradigms in which the behavioral and neural correlates of breathwork‐induced effects can be studied easily.

To date, most studies investigating the effects of breathing practice have focused on one or two specific practices. This limits comparability across studies and leaves open the question of whether any observed effects are specific to one particular breathing practice, for example, changing breathing rate of I/E ratio, or reflect broader effects tied to active or conscious respiratory control in general, that is, regardless of the specific pattern of respiration. In fact, it often remains unclear whether the observed behavioral benefits originate from directing attention towards the act of breathing itself, independent of the specific respiratory pattern, or whether they are linked to distinct temporal characteristics of the applied pattern.

The present study was designed to address these gaps by systematically isolating two key dimensions of structured respiration, namely, respiration frequency and I/E ratio. Specifically Experiment 1 focused on the changes in respiratory frequency by comparing slow and fast breathing, whereas Experiment 2 examined whether selectively prolonging inhalation or exhalation exerts differential effects on cognitive performance. By using short‐term interventions of 1 min across both experiments, we aimed to determine whether even brief manipulations of respiration are sufficient to alter sensory‐cognitive processing.

To probe the effects of short‐term breathwork, we relied on a well‐controlled sensory‐cognitive paradigm in the form of an emotion discrimination task. Previous work has shown that behavioral performance, that is, RTs and the fraction of correct responses (FCRs), vary systematically with respiratory phase (Harting et al. 2025; Johannknecht and Kayser 2022; Zelano et al. 2016). While this respiration–behavior coupling may arise via neural activity in the limbic system (Zelano et al. 2016), systematic studies also suggest that similar respiration–behavior coupling also emerges for other perceptual discrimination tasks (Brændholt et al. 2025; Johannknecht and Kayser 2022; Harting et al. 2025). Across the two experiments, we then contrasted the effect of different breathing techniques on the overall performance in this emotion discrimination task. In addition, we also performed a time‐resolved analysis of the behavioral data, as it remains unclear whether short‐term breathing practice alters perception, and if so, how long such effects persist.

## 2 Methods

A total of 63 participants took part in the study. The number of participants was based on previous studies studying benefits of breathwork (Chalaye et al. 2009; De Couck et al. 2019; Karjalainen et al. 2025; Rahmathulla et al. 2024). All procedures were approved by the ethics committee of Bielefeld University, and participants provided written informed consent prior to participation. All participants reported normal or corrected‐to‐normal vision. Participants were recruited from a typical student population via an online recruit system. Participants for the two experiments described below were recruited independently. They were selected from an available list of regular study participants in experiments performed in our lab and from spontaneous applications not recruited via a digital tool. The data were collected anonymously, and demographic data were not collected. Based on the participant pool, we expect the demographic characteristics of the sample to be broadly comparable with those reported in previous studies conducted in our laboratory (Park and Kayser 2021, 2022), with participants typically ranging from 20 to 40 years of age and a relatively balanced representation of both sexes. As participants were recruited independently for the two experiments, it is possible that some individuals participated in both experiments. This potential overlap should be considered when interpreting the results. However, as the statistical analyses for the two experiments were performed separately, potential overlap between the participant samples is not expected to affect the conclusions.

While participants were informed about the general aim of the study—to explore the relation between breathing practice and cognitive performance—they were not specifically informed of how their specific responses or RTs would be analyzed. In addition, participants did not receive specific information regarding the different breathing conditions or the study hypotheses. The experimenter was aware of the study purpose.

The experiments were conducted in acoustically shielded booths (E: Box; Desone, Germany). Respiratory data were measured using a thermistor (Littlfuse Thermistor No. GT102B1K, Mouser Electronics) inserted in a disposable medical oxygen mask, which covered the participants' mouth and nostrils, as described previously (Harting et al. 2025; Johannknecht and Kayser 2022; Stetza et al. 2025). The thermistor captured temperature fluctuations associated with inhalation and exhalation. The voltage drop across the thermistor was amplified in a custom‐made circuit and recorded via ActiveTwo EEG system (BioSemi BV) with a sampling rate of 1024 Hz. Visual stimuli and task instructions were presented via a projector using MATLAB (v. R2017a; The MathWorks Inc., Natick, MA) and the Psychophysics Toolbox (v. 3.0.18).

### 2.1 Experimental Paradigm

The behavioral data (RTs and response accuracy) were obtained in two separate experiments employing two very similar variations of an emotion discrimination task derived from previous work (Johannknecht and Kayser 2022; Harting et al. 2025). Although the paradigm has been frequently used to examine perceptual and affective processes, we do not expect any respiration–behavioral coupling to be specific to this paradigm as two systematic studies have revealed a similar coupling between RTs, response accuracy, and respiration across a larger sample of sensory detection and discrimination paradigms also involving very simplistic and abstract stimuli (Johannknecht and Kayser 2022; Harting et al. 2025).

Each trial started with a fixation period (0.7–1 s, uniform) followed by the presentation of a face showing one of two emotional expressions (duration of image presentation 0.16 s, covering 11.8° of visual angle). The images were taken from the FACES database (Ebner et al. 2010). For the experiment, we selected for 100 individuals two images for each emotion used in the experiments. Within a given block, each image was presented only once, ensuring no repeated exposure within blocks. Participants responded using the left or right arrow key on a computer keyboard. Intertrial intervals consisted of a fixed component of 1200 ms and a random component of up to 300 ms. In Experiment 1, 45 participants classified the emotions anger and disgust across three blocks per technique of 133 trials each. In Experiment 2, 18 participants classified the emotions happiness and sadness across two blocks per technique of 100 trials each. Prior to the experiment, we recorded each participant's spontaneous respiratory frequency for a duration of 2 min to establish an individual baseline frequency. Prior to each block of the emotion task, and after half the trials of each block, participants performed one of the breathing techniques described below for 60 s.

### 2.2 Respiratory Training

Prior to the experimental session, participants received standardized verbal and visual instructions on each of the designated breathing techniques. To ensure proper execution, each technique was once practiced for 1 min under the supervision of the experimenter. The choice of a 1‐min breathing practice was guided by the aim to model a brief intervention that reflects typical real‐world use (e.g., taking a few slow breaths before or during demanding situations) while also ensuring participants safety and tolerability as patterns with high frequencies or short expiration periods can lead to discomfort including dizziness or lightheadedness especially in inexperienced participants (Migliaccio et al. 2023). Participants were instructed to breathe exclusively through their nose during the structured breathing periods and were informed that they could resume their natural pattern immediately following the breathing practice. The breathing practice was guided by a continuous visual cue presented on a computer screen: a dynamically animated circle that expanded during the inhalation phase and contracted during the exhalation phase, providing a clear, time‐synchronized pacing signal (min. visual angle: 1.5°; max. visual angle: 5.4°). During the actual experiment, participants performed the breathing practice following these instructions but without direct supervision by the experimenter. The order of breathing practices was randomized in both experiments to minimize systematic order effects. We analyzed the characteristics of participants respiration during these practice periods to evaluate the adherence to the prescribed breathing practices (cf. Section 3.1).

In Experiment 1, we compared two breathing practices manipulating the frequency of respiration: a slow breathing technique, implemented as box breathing, and a fast breathing technique. Slow (box) breathing consisted of four sequential phases of equal duration: inhalation, breath hold, exhalation, and another breath hold. To individualize the duration of each phase, participants completed a standardized breath‐hold test prior to the breathing practice. This procedure was chosen based on evidence suggesting that breath‐hold time is positively associated with vital lung capacity and serves as an indirect measure of peripheral chemoreflex sensitivity (Cherouveim et al. 2013; Trembach and Zabolotskikh 2017). Based on each participant's breath‐hold performance, the durations of each phase were set to either 3 (breath‐hold time less than 20 s) or 5 s (breath‐hold time between 20 and 45 s), resulting in an overall respiratory cycle frequency of 0.08 or 0.05 Hz, respectively. For the fast technique, the respiratory frequency was set at twice the participant's baseline respiratory frequency. The respiratory cycle consisted of alternating inhalation and exhalation phases without periods of breath hold. Based on the definition for Tachypnea (increased or elevated respiratory frequency, Davis and Fisicaro 2016), doubling the spontaneous respiratory frequency should be sufficient to increase respiratory rate beyond the range covered by natural variation at rest, without at the same time mimicking very high frequency breathing that can be aversive. Normal resting respiratory frequency in healthy adults is typically reported to range between 12 and 20 breaths per minute (Anderson et al. 2010; Guijt et al. 2007). Accordingly, doubling the spontaneous respiratory frequency ensured that participants exceeded 20 breaths per minute, a threshold that has been used in previous studies as a minimum criterion for tachypnea (Bianchi et al. 2013; Puskarich et al. 2017), while ensuring that respiratory frequency was reliably increased in every participant.

In Experiment 2, we focused on a comparison of breathing practices altering the I/E ratios in both directions. While two conditions altered I/E ratios (short inspiration–long expiration [SILE] and long inspiration–short expiration [LISE]) (Röttger et al. 2021; Strauss‐Blasche et al. 2000), a no‐intervention condition was used as control condition (NOI). In SILE, inspiration and expiration were paced following a 1:2 ratio, emphasizing prolonged expiration, while in LISE, the ratio was inverted and emphasized prolonged inhalation. For both, the overall respiratory frequency was matched to each participant's spontaneous respiratory frequency. The no‐intervention condition contained no specific practice. For this, prior to the onset of the block, participants remained seated in the experimental room for 1 min, engaging in spontaneous, self‐paced breathing. As during blocks with actual breathing techniques, participants initiated the no‐intervention blocks by pressing a button. However, instead of a breathing exercise, they were presented with a 60‐s idle time before the first trial. For both experiments, we randomized the order of techniques (Figure 1).

**FIGURE 1:**
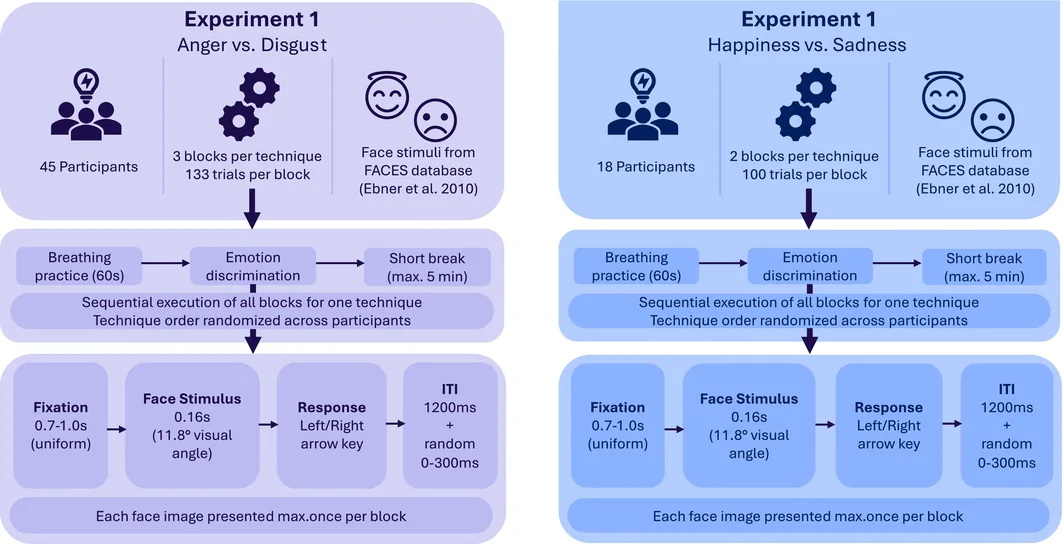
Experimental procedure for Experiments 1 and 2. Schematic overview of the experimental design and trial sequence. Details of the number of participants, blocks, trials, breathing practice, face‐stimulus presentation, response period, and intertrial interval are shown for each experiment.

### 2.3 Preprocessing of Respiratory Data

The respiratory data were preprocessed similarly as in our previous work (Harting et al. 2025; Stetza et al. 2025). First, the data were filtered using a third‐order Butterworth bandpass filter with cutoff frequencies of 0.03 (high pass) and 6 Hz (low pass). The filtered data were then resampled to 100 Hz using the FieldTrip toolbox (fieldtrip‐20231015). To facilitate interparticipant comparisons, the respiratory signal was normalized to *z* scores. The Hilbert transform was applied to derive the analytic signal, from which local peaks were identified. Individual respiratory cycles were extracted from 7‐s windows centered on each peak, retaining only those peaks for further analysis where the *z*‐scored signal exceeded a threshold of *z* = 0.5. Inhalation phases were defined as segments with a continuous positive slope prior to each peak, whereby interruptions shorter than 500 ms were interpolated. Exhalation phases were similarly defined as phases with a continuous negative slope following each peak, again interpolating interruptions shorter than 500 ms. Atypical respiratory cycles were identified by computing mean‐squared distances between individual cycles and the participant‐specific centroid of all cycles. Cycles with distances exceeding three standard deviations (SDs) from the centroid were classified as atypical. Atypical cycles may reflect breath holds, sighs, or clearing of the throat (Figure 2).

**FIGURE 2:**
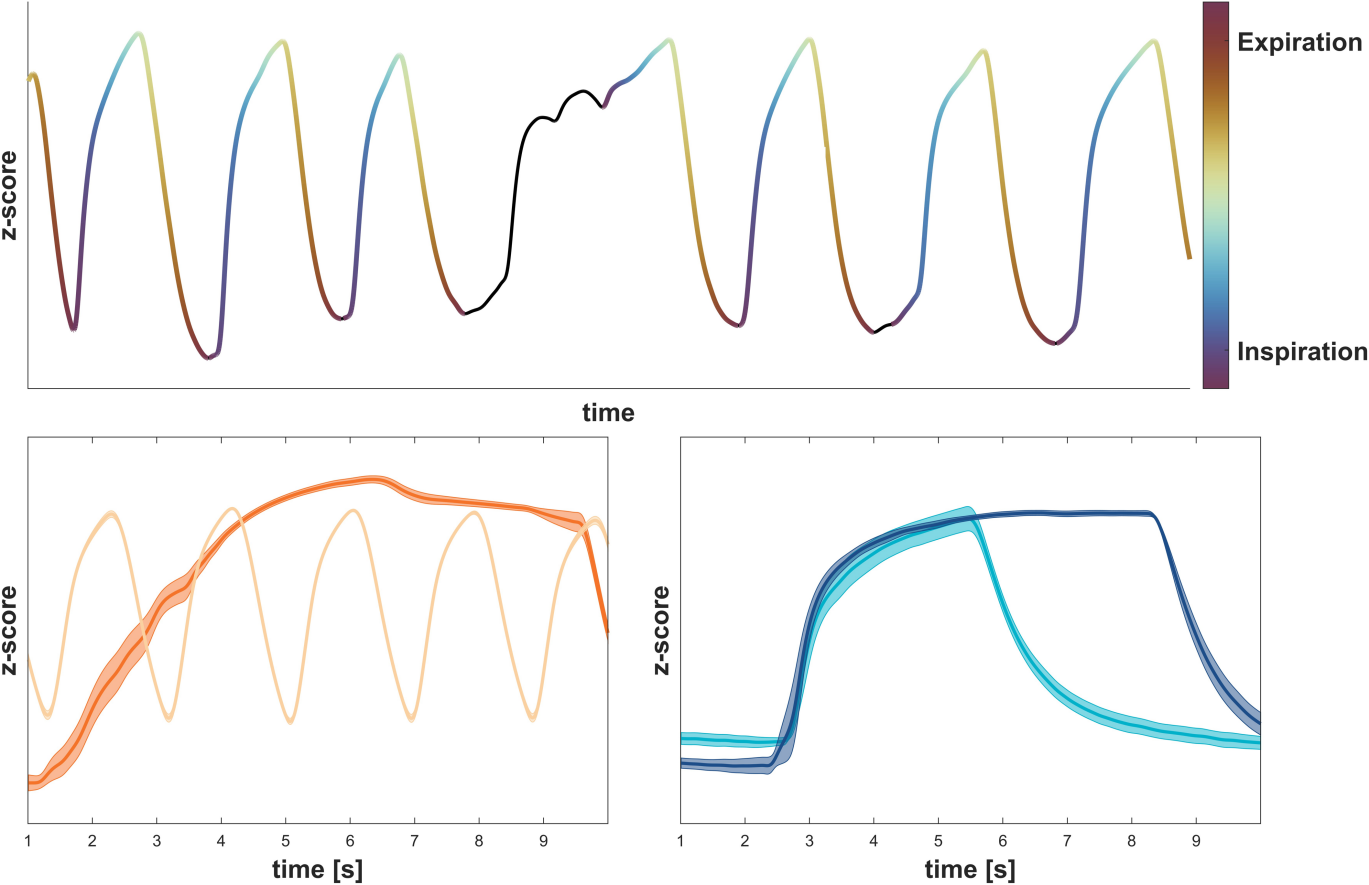
Exemplary respiratory traces illustrating spontaneous respiration and breathing practices. Each trace reflects the *z*‐scored respiration signal over time. Upper panel: Illustration of the phase classification applied to the respiration signal. The color scale reflects the respiratory phase. Undefined segments are marked in black. Lower left panel: Representative traces for the slow (box breathing, dark orange) and fast (hyperventilation, light orange) conditions from Experiment 1. Lower right panel: Representative traces for the SILE (light blue) and LISE (dark blue) conditions from Experiment 2.

### 2.4 Processing of Behavioral Data

The behavioral data for the emotion task comprised trial‐wise response accuracy and RTs. We removed a few individual outlier trials defined by the following criteria: Trials were excluded if RT was shorter than 200 ms or exceeded three times the median absolute deviation from the participant's individual RT distribution. This led to an exclusion of 4.91% ± 4.09% (mean ± STD) of trials in Experiment 1 and 5.29% ± 3.34% (mean ± STD) of trials in Experiment 2. For subsequent analysis, RTs were square root transformed and normalized within participants by subtracting the average, thereby controlling for individual differences in overall response speed. For the analysis of RTs, we focused only on trials with correct responses to isolate effects attributable to cognitive processing rather than motor errors or guessing. To quantify whether RTs follow a specific trend over trials following the respiration practice, we binned these in groups of 10 trials for each participant. We then used a linear regression to derive the slope of the binned RTs against the time passed in the experimental block.

### 2.5 Statistical Analysis

Our main analyses probed for systematic differences in task performance between techniques across participants. These tests were implemented separately for RTs and the FCRs using paired‐sample *t*‐tests. For each comparison, we report *p* values, Cohen's *d*′ as a measure of effect size, and Bayes factors (BF_10_) to quantify evidence for the alternative hypothesis that the mean difference deviates from zero. The significance level was set to *α* = 0.05. To control for multiple comparisons within the same experiment, *p* values were adjusted using the false discovery rate (FDR) correction method as proposed by (Benjamini and Yekutieli 2001). Note that *p* values reported through the manuscript are the corrected *p* values and hence can in principle exceed 1.

To test for a potential temporal trend in RTs following breathing practice, we used a permutation test. We compared the actual slope of the binned RTs against time passed in the experiment with a surrogate distribution of slopes obtained after randomly shuffling the binned RTs and calculating the slopes in 2000 permutations. From the surrogate distribution, we obtained the percentile *p* value of the original slope. Because the statistical analyses and sample sizes in the present study were not strictly planned based on previous results and effect sizes, we consider the study to be more explorative than confirmative.

AI‐assisted language editing was performed using OpenAI to improve clarity and grammar. No AI tools were used for data analysis or scientific content generation.

## 3 Results

### 3.1 Respiration During the Practice Period

Across two experiments participants completed four types of breathing practice during 1‐min breathing practice blocks prior to performing an emotion discrimination task. To characterize how participants breathed during the practice periods, we calculated their respiratory frequency and the I/E ratio and compared these with their baseline data (Figure 3).

**FIGURE 3:**
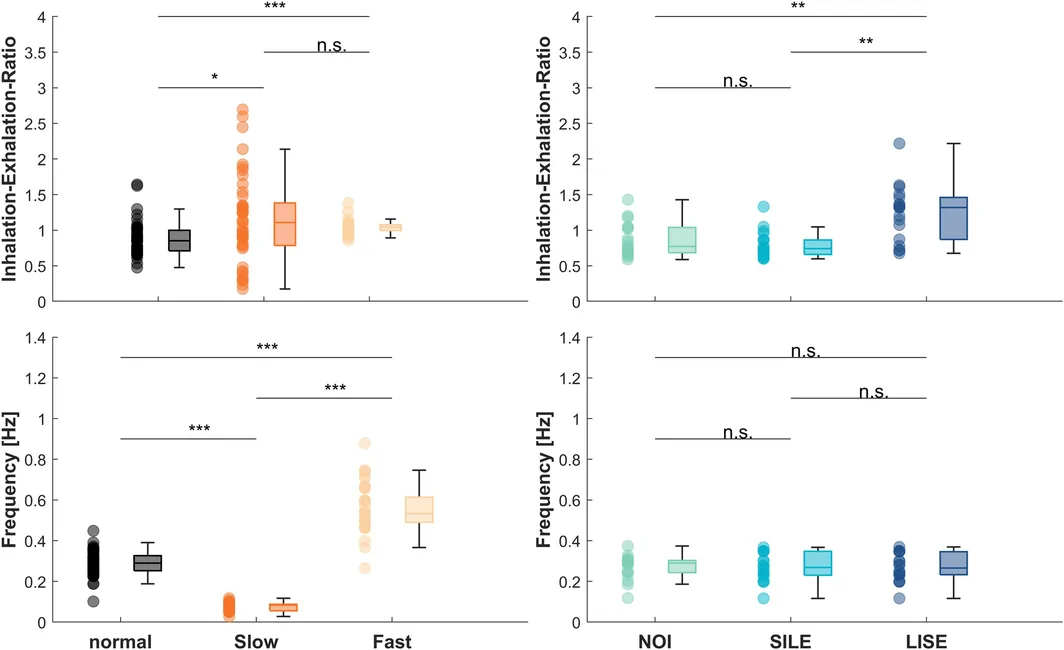
Respiratory characteristics during breathing practice for Experiment 1 (left panels) and Experiment 2 (right panels). The upper graphs show the comparison of inhalation‐to‐exhalation (I/E) ratio and the bottom graph those for respiratory frequency during the 1‐min breathing practice (Slow = slow‐paced breathing; Fast = fast‐paced breathing; SILE = short inspiration–long expiration; LISE = long inspiration–short expiration). Baseline values were obtained from either a baseline recording prior to the experiment (Experiment 1, “normal”) or from a 1‐min no‐intervention condition (Experiment 2, “NOI”). Data are presented as boxplots with individual participant values presented as dots. Statistical significance was determined using paired‐sample *t*‐tests; *p* values were adjusted for multiple comparisons using the false discovery rate (FDR). Asterisks indicate statistically significant differences: *p < 0.05; **p < 0.01; ***p < 0.001; n.s.: nonsignificant.

To facilitate the interpretation of the following results, G*Power (Faul et al. 2009) was used to determine the effect sizes associated with a statistical power exceeding 95% for two‐tailed *t*‐tests with an alpha level of 0.05. For Experiment 1, with a sample size of 45, a statistical power greater than 95% is reached for effect sizes exceeding Cohen's *d* = |0.550|, while for Experiment 2 (*n* = 18), a statistical power greater than 95% is achieved for effect sizes exceeding Cohen's *d* = |0.902|

In Experiment 1, participants (*n* = 45) followed either a slow or a fast breathing practice. During these periods, we observed clear and expected differences in respiratory frequencies: Slow breathing elicited markedly lower respiratory frequencies compared with fast breathing (*p* < 10^−5^, BF_10_ = 10^27^, Cohen's *d*′ = 5.9). Compared with baseline, fast breathing resulted in higher (*p* < 10^−5^, BF_10_ = 10^25^, Cohen's *d*′ = 2.9) and slow breathing in lower frequencies (*p* < 10^−5^, BF_10_ = 10^22^, Cohen's *d*′ = 4.6). As expected, the average I/E ratio did not differ significantly between slow and fast breathing (*p* = 0.523, BF_10_ = −3.6, Cohen's *d*′ = 0.2), but both ratios differed significantly compared with the baseline (slow: *p* = 0.021, BF_10_ = 5.0, Cohen's *d*′ = 0.6; fast: *p* < 10^−5^, BF_10_ = 499.1, Cohen's *d*′ = 0.9).

In Experiment 2, participants (*n* = 18) followed breathing practices aimed at manipulating the I/E ratio. As expected, we found no significant differences in respiratory frequency between conditions (NOI vs. SILE: *p* = 1.69, BF_10_ = −4.1, Cohen's *d*′ = 0; NOI vs. LISE: *p* = 1.69, BF_10_ = −4.1, Cohen's *d*′ = 0; SILE vs. LISE: *p* = 1.69, BF_10_ = −2.6, Cohen's *d*′ = 0). As expected, the breathing practices differed in their I/E ratios: The LISE condition exhibited a significantly higher ratio compared with the SILE condition (*p* = 0.007, BF_10_ = 31.4, Cohen's *d*′ = 1.4). Also, the I/E ratio in the LISE condition was significantly higher compared with participants' baseline ratio (*p* = 0.008, BF_10_ = 14.5, Cohen's *d*′ = 1.1). In contrast, no significant difference was observed between the SILE condition and baseline (*p* = 0.798, BF_10_ = −3.1, Cohen's *d*′ = 0.3).

This shows that participants were able to adhere to the structured respiration, resulting in marked differences in respiratory frequency or I/E ratio.

### 3.2 Respiration Following the Practice Period

Inspecting the individual respiratory traces revealed that participants exhibited highly variable respiratory patterns following the breathing practice blocks (examples are shown in Figure 4). Although explicitly instructed to return to natural, self‐paced breathing after the practice, the respiration data did not necessarily reveal such a return. We observed three main patterns across participants: some participants continued to breathe similar as during the breathing practice for a prolonged time, while others quickly reverted to a respiratory pattern as observed during their baseline. Moreover, some participants showed extended breath holding, sighs, or very atypical respiratory cycles that could not be classified based on the recorded signal. The boundaries between these postpractice patterns appeared fluid, with some participants gradually transitioning between them or displaying different respiratory responses when repeating the same breathing practice.

**FIGURE 4:**
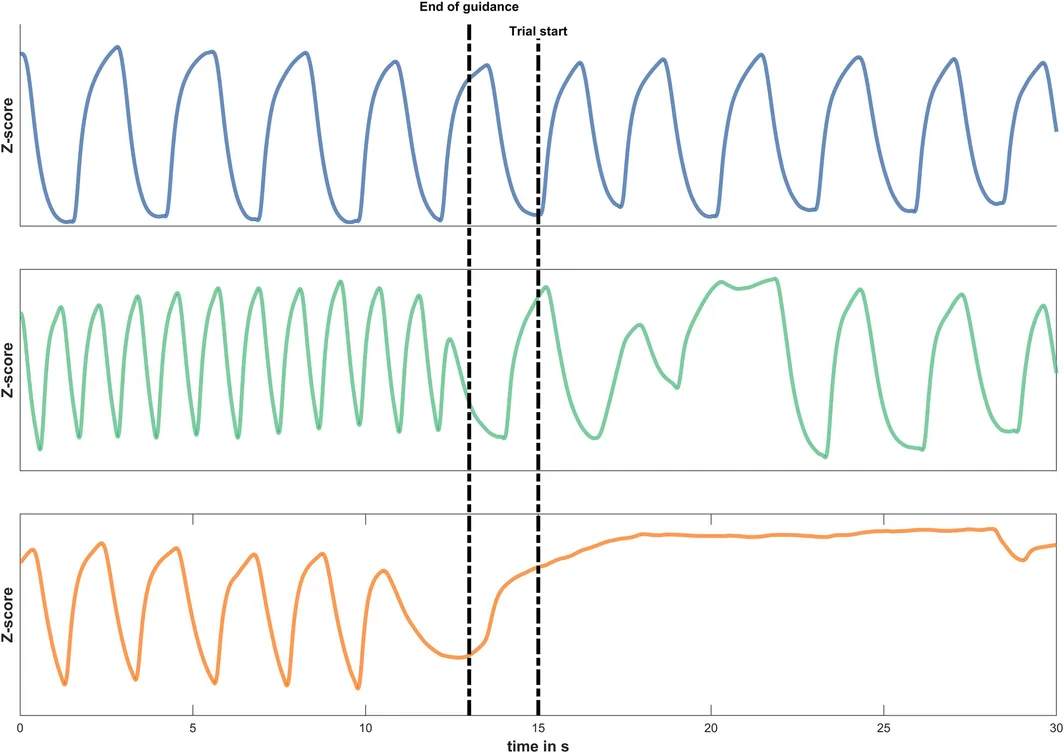
Exemplary respiratory traces from three participants illustrating individual differences in respiration following 1 min of fast breathing practice. Each trace displays the *z*‐scored respiratory signal over time (in seconds), with the vertical black lines indicating the end of the guided breathing practice and task onset (2 s after the end of guided breathing practice). The top example continues to breathe as during the breathing practice, the middle quickly returns to baseline, and the bottom displays some forms of breath hold or extended sigh. Note that the frequency for hyperventilation was dependent on each participant's baseline frequency.

To examine whether atypical respiratory patterns were associated with specific breathing practices, we calculated the proportion of data points that could not be assigned to a specific respiratory phase (inhalation or exhalation) within 1 min following the end of guided breathing practice. Cycles containing a high amount of undefined data points may for example reflect breath holds, sighs, or otherwise unstructured breathing. For remaining cycles, we calculated the frequency and the I/E ratio (see Figure 5).

**FIGURE 5:**
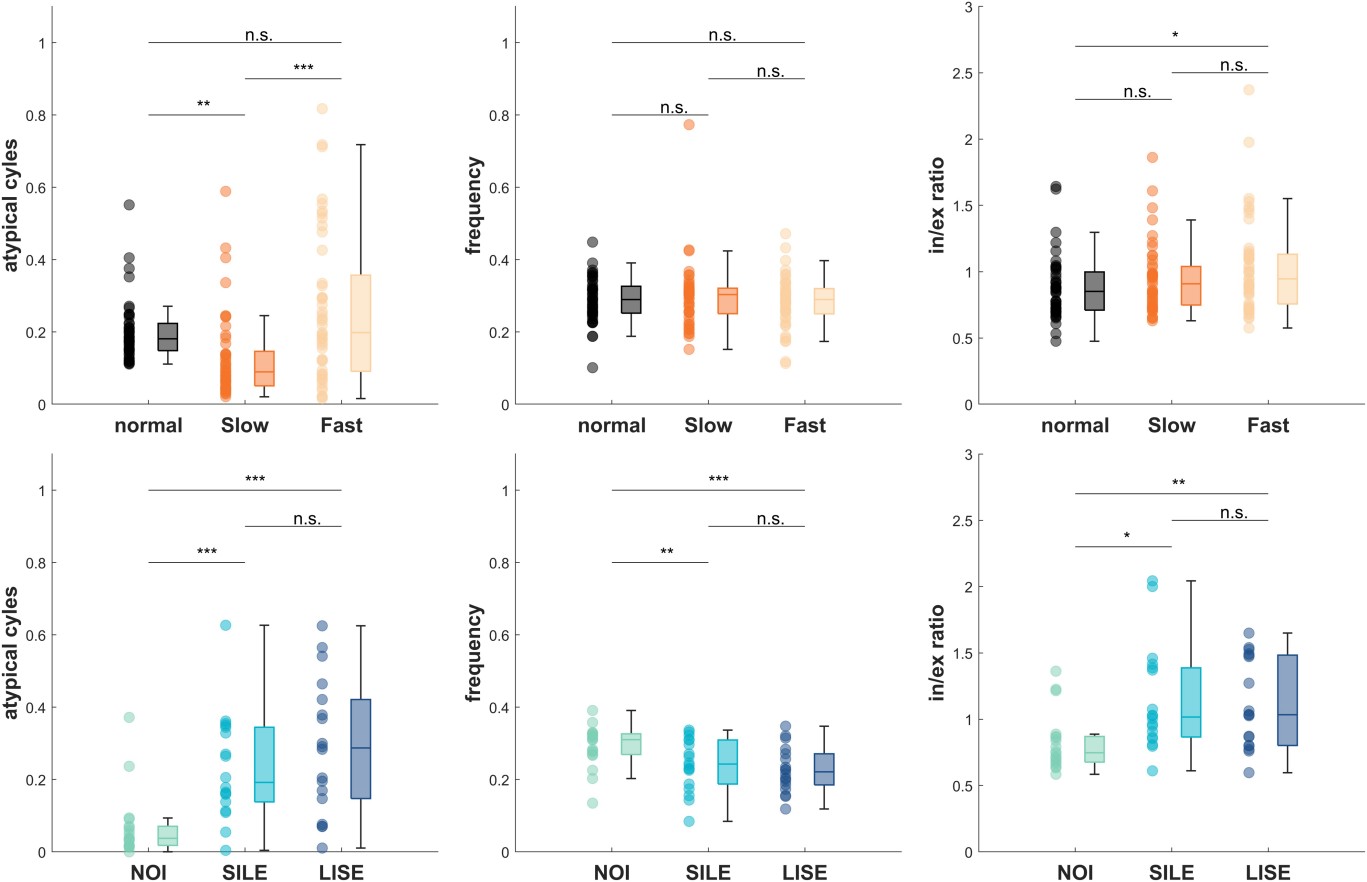
Respiration characteristics in a period of 1 min following the breathing practice. The upper row presents results from Experiment 1 (slow and fast breathing) and the bottom row from Experiment 2 (SILE, LISE, and NOI). The left panels show the percentage of undefined data points per participant (see Section 2); the middle panels depict the respiratory frequency; the right panels illustrate the inhalation‐to‐exhalation ratio of those cycles that could be defined by the segmentation algorithm. Data are visualized using boxplots, with individual participant data shown as dots. Statistical comparisons were performed using paired‐sample *t*‐tests using the false discovery rate (FDR) to correct for multiple comparisons. Asterisks indicate statistically significant differences: *p < 0.05; **p < 0.01; ***p < 0.001; n.s.: nonsignificant.

In Experiment 1, the percentage of undefined data points differed significantly between the fast and slow breathing conditions (*p* < 10^−5^, BF_10_ = 4165.9, Cohen's *d*′ = −0.8), with a higher proportion observed after fast breathing. After the application of the slow breathing practice, the proportion of unclassified data points was reduced compared with the baseline condition (normal vs. slow: *p* = 0.001, BF_10_ = 52.1, Cohen's *d*′ = 0.6). In contrast, the fast breathing practice elicited no statistically significant reduction or increase in undefined datapoints (normal vs. fast: *p* = 0.09, BF_10_ = 1.0, Cohen's *d*′ = −0.38). There was no difference in respiratory frequency during the 1‐min block after the breathing practice between conditions (slow vs. fast: *p* = 1.150, BF_10_ = −4.5, Cohen's *d*′ = 0.2; normal vs. slow: *p* = 1.150, BF_10_ = −5.5, Cohen's *d*′ = −0.1.; normal vs. fast: *p* = 1.15, BF_10_ = −5.5, Cohen's *d*′ = 0.1). The I/E ratio differed between the recording of baseline respiration and the fast condition (*p* = 0.041, BF_10_ = 5.1, Cohen's *d*′ = −0.5) but not between the recording of baseline respiration and the slow condition (*p* = 0.095, BF_10_ = −1.0, Cohen's *d*′ = −0.3) or between the breathing practice conditions (*p* = 0.095, BF_10_ = −1.2, Cohen's *d*′ = −0.3) (Figure 5, upper row).

In Experiment 2, we manipulated the I/E ratio. Significant differences emerged in the percentage of undefined data points after performing SILE and NOI (*p* = 0.001, BF_10_ = 91.5, Cohen's *d*′ = −1.3), as well as between LISE and NOI (*p* = 0.001, BF_10_ = 141.8, Cohen's *d*′ = −1.4). Respiratory frequency in the minute after both breathing practices differed from the no‐intervention condition (NOI vs. SILE: *p* = 0.003, BF_10_ = 32.1, Cohen's *d*′ = 0.7; NOI vs. LISE: *p* = 0.0003, BF_10_ = 508.5, Cohen's *d*′ = 1.0). Moreover, a significant difference in the I/E ratio was observed after SILE and LISE practice in comparison with the no‐intervention condition (NOI vs. SILE: *p* = 0.020, BF_10_ = 6.8, Cohen's *d*′ = −1.0; NOI vs. LISE: *p* = 0.007, BF_10_ = 32.2, Cohen's *d*′ = −1.0). Statistically there was no difference between SILE and LISE condition, neither for the frequency (*p* = 0.406, BF_10_ = −2.1, Cohen's *d*′ = 0.2) nor for the I/E ratio (*p* = 1.412, BF_10_ = −4.0, Cohen's *d*′ = 0.1) in the minute after breathing practice (Figure 5, lower row).

Overall, fast, SILE, and LISE breathing practice exhibited a recovery of spontaneous respiration that differed from that observed following slow respiration. Hence, slow breathing may specifically enable a fast and structured return to a spontaneous and regular respiratory pattern compared with the other techniques.

### 3.3 Impact of Respiration Training on Task Performance

We analyzed the FCRs (Figure 6) and RTs (Figure 7) following each breathing practice. When considering the data from the entire experimental blocks, we found no significant differences between breathing techniques in either Experiment 1 nor Experiment 2. The Bayes factors associated with individual comparisons were mostly in favor of no effect (indicated here by negative Bayes factors) but reflect either anecdotal or moderate evidence (Table 1).

**FIGURE 6:**
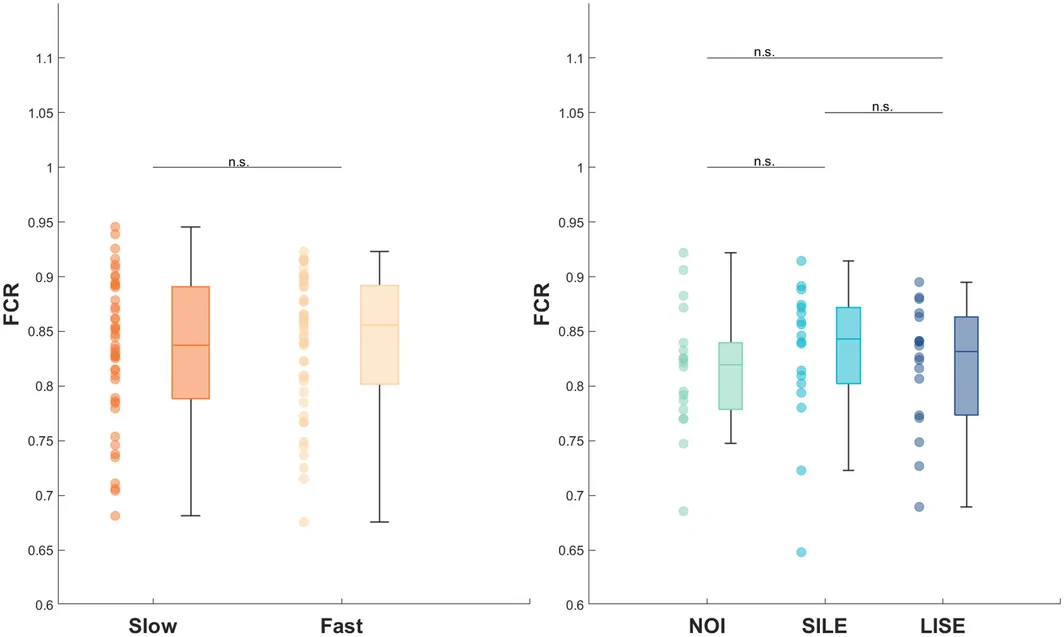
Response accuracy following breathing practice. Average fraction of correct responses (FCRs) for each breathing practice. The left panel shows data from Experiment 1 (slow and fast breathing), and the right panel shows data from Experiment 2 (NOI, LISE, and SILE). The data are displayed as boxplots with individual data shown as dots. Pairwise statistical comparisons were performed using *t*‐tests, with *p* values corrected for multiple comparisons using the false discovery rate (FDR). Asterisks indicate statistically significant differences: *p < 0.05; **p < 0.01; ***p < 0.001; n.s.: nonsignificant.

**FIGURE 7:**
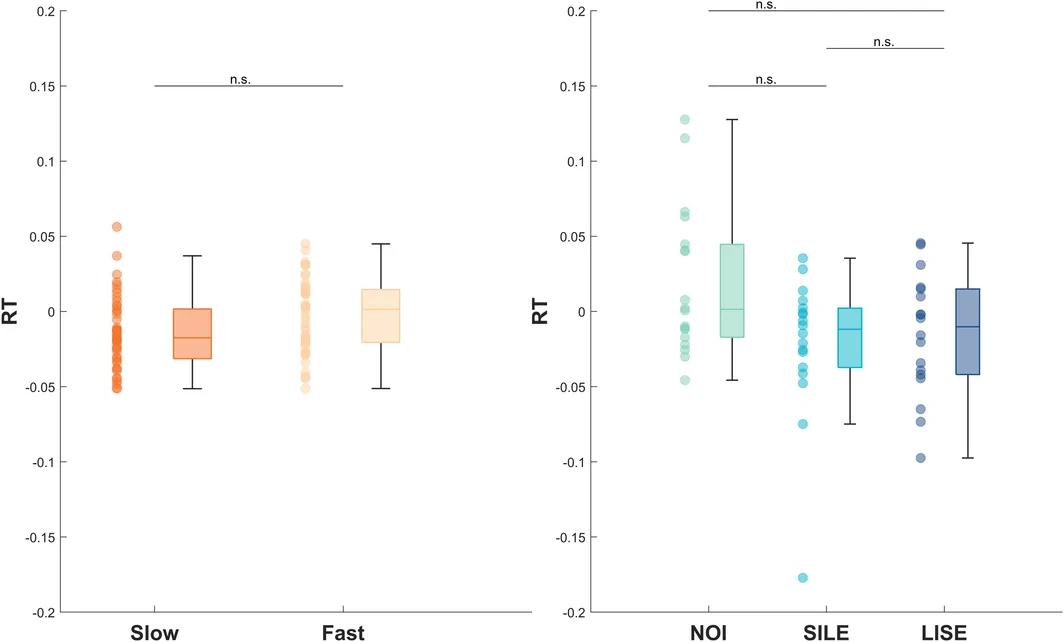
Reaction times (RTs) following breathing practice. Distribution of average RTs for each breathing condition. The left panel shows the results from Experiment 1 (slow and fast breathing) and the right panel from Experiment 2 (NOI, LISE, and SILE). For each participant, mean RTs were computed from all valid trials within the respective task block (see Section 2). Data are visualized using boxplots, with individual participant data shown as dots. Statistical comparisons between conditions were performed using paired‐sample *t*‐tests, and *p* values were adjusted for multiple comparisons using the false discovery rate (FDR). Asterisks indicate statistically significant differences: *p < 0.05; **p < 0.01; ***p < 0.001; n.s.: nonsignificant.

**TABLE 1:** Results of paired‐sample *t*‐tests comparing reaction times (RTs) and fraction of correct responses (FCRs) between breathing conditions in Experiment 1 and Experiment 2.

	FCR	RT
Slow vs. fast		
*p* value	0.199	0.095
BF_10_	−2.8	−1.6
Cohen's *d*′	0.1	0.5
NOI vs. SILE		
*p* value	1.333	0.200
BF_10_	−2.4	1.2
Cohen's *d*′	0.2	0.8
NOI vs. LISE		
*p* value	1.333	0.200
BF_10_	−3.3	−1.1
Cohen's *d*′	0.1	0.7
SILE vs. LISE		
*p* value	1.564	1.293
BF_10_	−4.1	−3.9
Cohen's *d*′	0.2	0.1

*Note:* Reported are *p* values (corrected for multiple comparisons using the false discovery rate [FDR]), together with BF_10_ and Cohen's *d*′ for each pairwise comparison.

While the overall effects of breathing practice seem to be small, visual inspection of the data revealed that there could be shorter lived effects immediately following the practice. To quantify such trends in behavior, we binned the experimental trials into bins of 10 trials. We then quantified whether RTs or FCR systematically change over the course of the experimental block using a regression analysis combined with a permutation test.

The results show that for all breathing practices, the slopes of any trends in RTs differed significantly from the null hypothesis of no significant slope. Notably, for all breathing practices, these slopes were positive, indicating a progressive slowing of RTs over time after the application of breathing practice. Importantly, RTs during the NOI condition did not show a slope differing from 0, suggesting that this dynamic of RTs (starting faster in a block and then slowing down) may be specific to having performed some form of breathing practice and not a general effect of fatigue during each block (see Table 2 and Figure 8). To directly corroborate this conclusion, we compared the slopes between conditions in Experiment 2. This revealed a significantly higher slope for SILE versus the NOI condition (*p* = 0.015, BF_10_ = 3.7, Cohen's *d*′ = 0.8) but not for LISE (*p* = 0.152, BF_10_ = −1.6, Cohen's *d*′ = 0.4).

**TABLE 2:** Results of randomization test for the linear regression analysis of reaction times (RTs) and fraction of correct responses (FCRs) across trials.

	RT	FCR
Slow		
Percentile	99.99	3.45
Original slope	0.0064	−0.007
Fast		
Percentile	99.99	0.13
Original slope	0.0064	−0.011
NOI		
Percentile	59.15	72.21
Original slope	0.0012	0.0067
SILE		
Percentile	100	6.25
Original slope	0.018	−0.01
LISE		
Percentile	99.90	93.42
Original slope	0.010	0.012

*Note:* The table reports *p* values derived from the permutation procedure, indicating whether the slope of the regression fitted to the original data differed significantly from slopes obtained from surrogate data. For each condition, the original regression slope is provided.

Abbreviations: Fast = fast breathing; LISE = long inspiration–short expiration; NOI = no intervention; SILE = short inspiration–long expiration; Slow = slow breathing.

**FIGURE 8:**
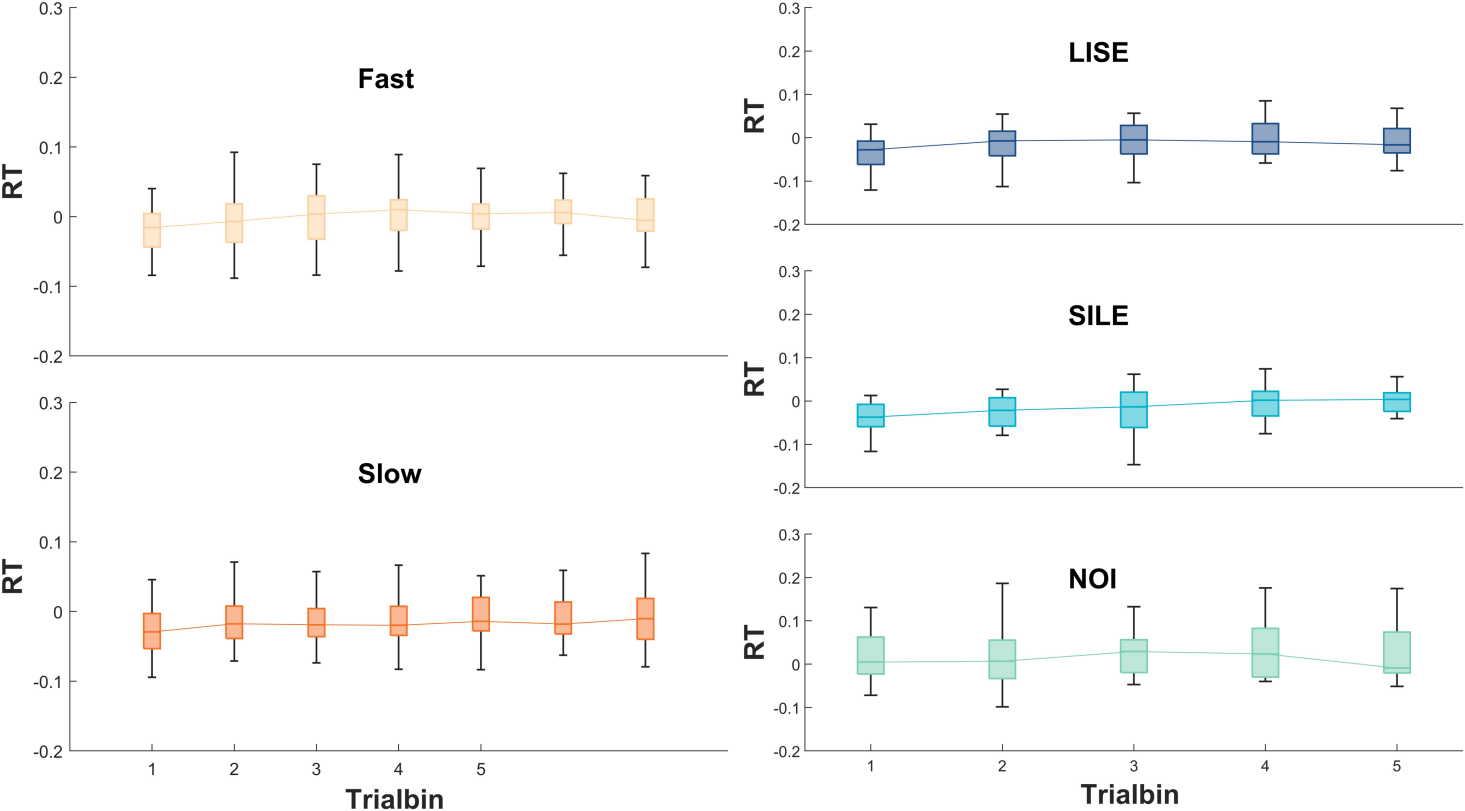
Temporal dynamics of RTs across trial bins for each breathing condition. The figure displays the binned reaction times (RTs) during the experimental blocks. Each panel shows the data for one breathing condition, with the two panels on the left corresponding to Experiment 1 (slow and fast breathing) and the three panels on the right to Experiment 2 (LISE, SILE, and NOI). For each participant, the RT per bin was computed as the mean RT of all trials within that bin after excluding trials based on criteria described in Section 2. Boxplots represent the distribution of normalized and squared RTs across participants. Results of the permutation tests assessing the significance of the linear slope across bins, as well as the original slope of the fitted regression line, are reported in Table 2.

For the FCRs, this analysis revealed a significant negative slope for the fast breathing condition (percentile in random permutation test: 0.13; slope: −0.011), indicating a decline in accuracy over time. In contrast, for all other breathing techniques and the NOI condition, the slope of the linear regression did not significantly differ from zero, suggesting no systematic change in accuracy across trials (see Table 2 and Figure 9).

**FIGURE 9:**
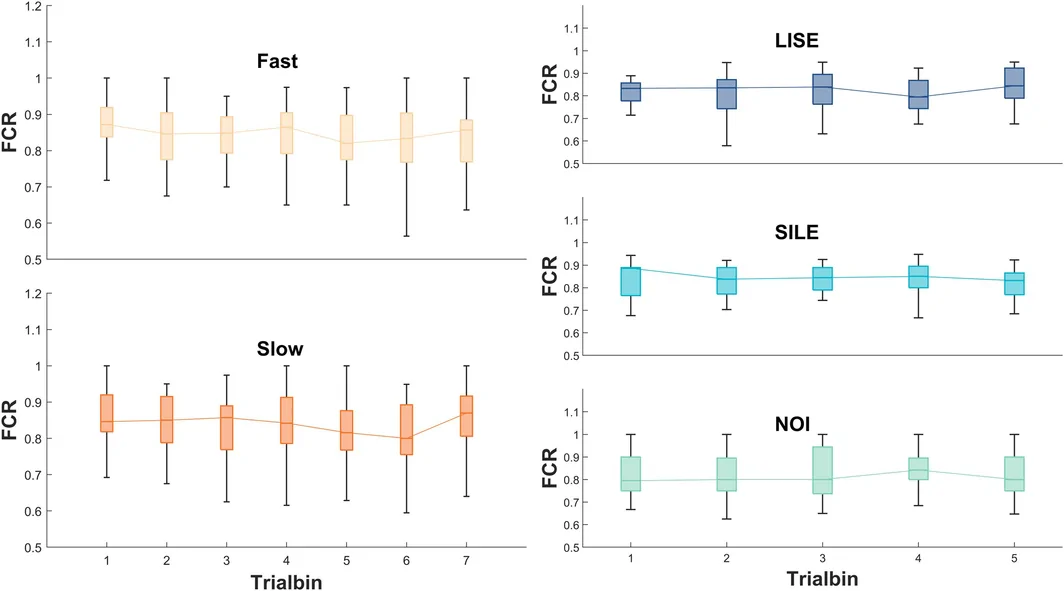
Temporal dynamics of task accuracy (FCR) across trial bins for each breathing condition. The figure illustrates changes in participants' accuracy—indexed by the fraction of correct responses (FCRs)—over the course of the postpractice task. Data are binned into segments of 10 consecutive trials for each participant. The FCR per bin was calculated as the proportion of correct responses among valid trials (see Section 2). The two panels on the left correspond to Experiment 1 (slow and fast breathing) and the three on the right represent Experiment 2 (LISE, SILE, and NOI). Boxplots show the distribution FCR values across participants. The outcome of the permutation‐based slope analysis and the original slope values for each breathing condition are reported in Table 2.

## 4 Discussion

### 4.1 Adherence to Guided Breathing Practice and Postpractice Respiratory Dynamics

Our results demonstrate that participants were generally able to adhere to the instructed pattern of respiration. At the group level, these short‐guided breathing protocols induced the expected changes in respiratory rate and I/E ratio. Particularly for the fast and slow breathing practice, participants breathed significantly faster (slower) than during spontaneous respiration, indicating the successful implementation of the externally paced respiration. For the techniques manipulating the I/E ratio, we observed that the individual respiratory patterns during the breathing practice were more heterogeneous. Several participants exhibited I/E ratios opposing the intended pattern, highlighting potential difficulties in adhering to the instructed patterns. This highlights the importance of monitoring individual respiratory performance in studies investigating the impact of breathing practice.

When inspecting participants' patterns of respiration in the minute immediately after the breathing practice, we observed an increased prevalence of atypical respiratory cycles compared with recordings of spontaneous respiration or conditions without a breathing practice. The emergence of such atypical cycles suggests that some participants' required time to return to their typical respiratory pattern after the practice of conscious breathing and possibly followed the practice period with prolonged breath holds, sighs, or other untypically structured respiratory cycles. We can only speculate about why this is the case. One possibility is that the application of respiration practice alters the physiological homeostatic balance (e.g., partial pressures of oxygen and carbon dioxide in the blood). The more this homeostatic balance is displaced from equilibrium, the stronger the compensatory response required to restore this to baseline. Alternatively, this reflects participants' general flexibility in quickly adjusting their respiratory pattern. For sure, the individual variability in how accurately participants follow the instructed breathing practice and how they breath following this requires more attention in future work.

The pattern of respiration following the breathing practice may also be critical for understanding what specific effects or how strong an effect of the specific practice actually is. The variability in respiratory patterns observed following guided breathing raises the possibility that differences in the respiratory characteristics following breathing practice contribute to the heterogeneous effects across participants. Continuing to breathe similar to the instruction may amplify effects, while a quick return to baseline may reduce them in comparison. The influence of atypical cycles, such as breath holds or sighs, in turn remains unclear. This underscores the need to study participants' breathing behavior more systematically during and following guided breathing interventions.

### 4.2 Short‐Lived Cognitive Effects of Breathing Practice and Potential Underlying Mechanisms

Despite inducing systematic changes in participants respiration, we did not find systematic differences in participants behavioral performance (FCR or RTs) across the full postpractice blocks between the different breathing practices. Importantly, in Experiment 2, we also did not see significant differences between the respiration conditions and a dedicated no‐intervention manipulation. This speaks against longer lived and systematic benefits following structured 1‐min breathing practice, at least for the patterns and the task studied here.

However, a time‐resolved analysis revealed transient changes in RTs over the time course of an experimental block following the breathing practice. Effectively, RTs were shorter immediately following the breathing practice and became slower over the course of the testing block. Importantly, this effect was present for all breathing techniques but not following the no‐intervention condition. This suggests that even a brief period of structured respiration may enhance task responsiveness, although the benefit appears short lived and dissipates over the time scale of tens of seconds.

One interpretation of the transient enhancement in RTs is a short‐term shift in autonomic state. Although an influence via the HPA axis appears unlikely on the short time scale considered here, a potential involvement of this system cannot be entirely ruled out, given that studies on long‐term practice of deep breathing have demonstrated systematic changes in cortisol levels (Ma et al. 2017; Martarelli et al. 2011; Perciavalle et al. 2017). Alternatively, changes in the balance of the autonomic nervous system may emerge transiently following breathwork. Previous work has shown that slow‐paced breathing (De Couck et al. 2019), hyperventilation (Neginhal et al. 2017), and modified I/E ratio patterns (Van Diest et al. 2014) lead to changes in HRV. Given a link between vagal tone and cognitive control (Smith et al. 2017; Thayer and Lane 2000, 2009), this in turn may reflect changes in the autonomic nervous system through which breathing practice could indirectly modulate cognitive performance. Alternatively, or possibly complementary, respiration may directly modulate large‐scale brain activity. Recent studies have demonstrated that respiration and neural oscillations are tightly coupled during both rest and task engagement (Kluger and Gross 2021; Kluger et al. 2021; Zelano et al. 2016). These respiration‐locked modulations in oscillatory power have been shown to account for variations in task performance, suggesting that rhythmic changes in neural excitability may underlie respiration‐related fluctuations in perception and cognitive processing (Kluger et al. 2021). Such modulations of neural oscillations have also been observed during high‐frequency yoga breathing (Budhi et al. 2024), voluntary deep breathing (Karjalainen et al. 2025), and slow controlled breathing (Hsu et al. 2020). Transient modulations of cortical dynamics may provide a mechanistic explanation for the short‐lived behavioral improvements in RTs observed in the present study. Once the breathing practice ends and spontaneous respiration resumes, the induced neural state likely returns to baseline, which could account for the temporal limitation of the observed effects. However, there is currently little evidence regarding how different breathing practices, especially those manipulating the I/E ratio, affect oscillatory activity, representing an important open question for future research.

In the present study, a breathing practice duration of 1 min was chosen to ensure participants safety, while maintaining ecological validity with respect to real‐world applications. At this stage, the effects of longer breathing interventions on the aforementioned processes remain speculative. Nevertheless, it is plausible that extended practice durations would induce more pronounced deviations from the homeostatic balance, thereby prolonging the subsequent recovery phase. Such prolonged physiological modulation may, in turn, result in more sustained effects on cognitive performance measures, including response accuracy and RT. Future studies considering different durations of breathing practice could provide valuable insight into dose–response relationships.

### 4.3 Limitations and Outlook

The present study tested effects of breathwork on performance in a visual emotion discrimination task. We do not believe that the nature of the task has a direct implication on the null result. In fact, we would assume that any influence of breathwork facilitating sensory or cognitive performance would be visible across a range of abstract sensory tasks and tasks relying on identifying object structure or emotional valence. This reasoning is based on the observation that effects pertaining to respiratory alignment emerge similarly across a wide range of paradigms (Harting et al. 2025; Johannknecht and Kayser 2022). It is also supported by the assumption that one major influence of breathwork may be via regulating stress, arousal, or other forms of homeostatic balance, which should not be specific to certain types of sensory information. However, we also cannot rule out that the specific task had a decisive impact on the result, and clearly, further work is required to understand how and whether short‐term breathwork facilitates general sensory or cognitive processes.

The present study has several limitations. It is clearly explorative in nature and not strictly planned based on previously tested protocols or effect sizes. First, the duration of the breathing practice was short (1 min), which may not be sufficient to induce lasting and sustained changes in bodily physiology or the regulation of the autonomous nervous system. This short duration may explain the limited differentiation in effects in the cognitive task across techniques. Second, although we analyzed individual respiratory traces, we did not systematically monitor the physiological parameters such as heart rate, HRV, or respiratory gas composition (e.g., CO_2_ or O_2_ concentration). This makes it difficult to link the individual variability in respiratory patterns to the outcome. Third, the no‐intervention condition, in which participants sat quietly and breathed naturally, served as a reference for spontaneous respiration and physiological resting state rather than controlling for attentional engagement or task anticipation. It allowed comparison against structured breathing practice to ensure that observed effects were not driven by natural variability or temporal factors. Future studies should implement control conditions such as breath awareness or heartbeat counting tasks to isolate the effects of attentional focus on interoceptive signals from those attributable to the alteration of breathing patterns per se. Moreover, future work should consider paradigms targeting other sensory modalities such as audition or vision to verify whether observed effects generalize beyond the chosen paradigm. Finally, we did not measure brain activity (e.g., by using EEG) during or after the breathing practice. This limits our ability to link respiration‐induced behavioral changes to underlying neurophysiological mechanisms. Addressing these points in future research will be essential for more precisely disentangling the pathways through which respiratory practice influences cognitive function.

## 5 Conclusion

The present study demonstrates that even brief applications of guided breathing practices can transiently modulate cognitive performance, as reflected in faster RTs immediately following the practice. While the effects were short lived and may be independent of the specific breathing practice, they suggest a general facilitative impact of structured respiration on attentional readiness or sensorimotor responsiveness. These findings underscore the feasibility of implementing guided breathing in experimental paradigms, highlight the relevance of temporal dynamics when assessing their cognitive effects, and raise important questions about the underlying mechanisms.

## Author Contributions

**Lena Hehemann:** conceptualization, investigation, writing – original draft, methodology, visualization, writing – review and editing, software, formal analysis, project administration, data curation. **Lisa Stetza:** conceptualization, writing – review and editing, methodology, software. **Christoph Kayser:** conceptualization, writing – review and editing, supervision, investigation, software, project administration.

## Conflicts of Interest

The authors declare no conflicts of interest.

## Data Availability

The preprocessed data used for statistical analyses and generation of the figures, together with the code used for data analysis and visualization, are publicly available in the GitHub repository: https://github.com/LenaHehemann/Effects‐of‐Short‐Term‐Breathwork‐on‐Respiration‐and‐Cognitive‐Performance‐in‐a‐Discrimination‐Task. The original/raw datasets are not publicly available in their entirety but are available upon reasonable request.
